# New York Patholgoical Society

**Published:** 1879-08

**Authors:** 


### NEW YORK PATHOLOGICAL SOCIETY.

*Waxy degeneration of the placenta.*—Dr. C. Heitzman presented microscopic specimens prepared from six placentæ, which exhibited a change in placental tissue heretofore undescribed. In all the cases premature birth had taken place; the earliest at the fifth and the latest at the eighth month of uterogestation. To the naked eye, the placentæ exhibited grayish or yellowish patches scattered through the solid part, the decidua, and less marked through the villous portion.

In all the cases the diagnosis of fatty degeneration of the placenta had been made, and Dr. H. was of the same opinion when he first saw the specimens. In all, the size of the placenta was normal, corresponding to the month of pregnancy. In all, the fœtus was well developed.

In some of the cases the fœtus was dead at the time of its birth, and in those in which the child was born alive it died shortly after, breathing only a few times.

In no instance was there any clinical history of syphilis.

In a number of the cases the premature birth occurred in apparently perfectly healthy women.

The first specimen was a placenta that was presented to the New York Pathological Society by Dr. Salvatore Caro, as an illustration of the fatty degeneration, and taken from a woman who had had a succession of premature births dependent upon that cause.

Dr. Heitzmann had examined the specimen many times, and had failed each time to find any microscopical evidence of fatty degeneration, although the gross appearance was such as indicated its presence. The specimen had been preserved in chromic acid, a fluid not unfavorable to the perfect preservation of fatty tissue, and yet he had not been able to discover anything which he could call fatty degeneration. On the contrary, the microscope revealed a change that had not heretofore been described as existing in the placenta. There was found a peculiar homogeneous, shining material, which took carmine, was slightly stained with chloride of gold, and presented the ordinary appearance of waxy degeneration. There were no protoplasmic bodies present. In the villous portion the same change existed, but to a less marked degree. A large number of the capillaries were lost, being transposed into the same material. The myxomatous tissue of the placenta was missing altogether in the portions affected. This change Dr. Heitzmann had found in six specimens of so-called fatty degeneration of the placenta, and the question arose in his mind, Was there any such thing as fatty degeneration of the placenta? Was not the change, which had been regarded as fatty, a waxy degeneration?

In one case the fœtus was examined, and no evidence of fatty degeneration was found.

The report was presented as a provisional one.

Dr. C. C. Lee asked Dr. Heitzmann if he was able, from his researches, to suggest any therapeutical measures to prevent the change described?

Dr. Heitzmann replied that so long as we did not know what the essential chemical change was, we could not expect to reach any conclusion regarding its therapeutics. The microscope thus far had given but few practical facts with regard to therapeutics.

Dr. J. C. Peters referred to the fibrin-destroying function of the liver, and suggested it might be barely possible that, in those cases in which syphilitic contamination was not present, attention might be directed to that organ.

Dr. Heitzmann thought that fibrin did not have much to do with the change known as waxy degeneration.

Dr. J. W. Howe believed it fair to assume that other observers were correct in reference to what they had determined to be fatty degeneration of the placenta.

*Cystitis — Suppurative Nephritis — Perinephritic Abscess — Acute Peritonitis.*—Dr. C. C. Lee presented the urinary organs removed from a woman who died from acute peritonitis following perinephritic abscess, which in turn followed pyelitis and cystotomy:

S. J., æt 36, born in London, widow, had been ill for eighteen months with symptoms of cystitis. She had previously been treated for Bright's disease and anteflexion of the uterus. At times she had passed a large amount of pus with urine, which, at other times, was quite clear. No uræmic symptoms had ever occurred. She was admitted to the Woman's Hospital October 24, 1878. A long course of treatment for her cystitis produced no benefit, and February last she was transferred from Dr. Emmet's service to Dr. Lee's. At that time her bladder was contracted and so irritable as to hold only an ounce of urine. As all her previous treatment had failed to relieve it, he performed cystotomy February 12th, keeping the opening patent with a glass tube. In ten days the bladder symptoms improved greatly, but sweating, high temperature and chills began to appear and recurred regularly. Quinine was used freely without benefit. The tube was removed March 21st. In ten days from that date pain appeared in left renal region, and in two days this was followed by a circumscribed swelling, which indistinctly fluctuated. It was aspirated and pus was obtained. Aspirated twice again, and finally opened freely under spray; abscess washed out daily; drainage tube kept in; but the patient slowly sank, and died April 24, 1879.

Autopsy twenty-four hours after death. Body emaciated. There was an incision about two centimetres above the middle of the crest of the ilium. That incision extended through the abdominal walls into an abscess seated on the anterior face of the left kidney. The abscess had opened into the peritoneal cavity, so that a probe could be passed from the external opening directly into the peritoneal cavity, the internal opening being nearly opposite the external, being situated to the left of the descending colon, three centimetres above the crest of the ilium. The accumulation of pus in the abdominal cavity, which communicated with the perinephritic abscess, was encapsulated by recent adhesions between the lumbar and iliac regions. There were about eight ounces of pus thus shut in. There was also a moderate amount of general peritonitis, evidenced by recent fibrin on the visceral and parietal peritoneum. There was a small abscess in Douglas' cul-de-sac about the size of a pigeon's egg, situated on the right side and encapsulated by recent adhesions.

Heart normal; decolorized post-mortem clot in right ventricle.

Pleura: Extensive old pleuritic adhesions on right side Lungs normal.

Spleen normal.

Kidneys: The *left* kidney was changed into a sacculated mass: the cavities were filled with pus; numerous small abscesses were in the septa which separate the larger cavities; the pelvis was not dilated; on the other hand, it appeared contracted, and its mucous membrane thickened and coated with pus. There seemed to be no kidney parenchyma left; the capsule was thickened; there was a sloughy abscess in the connective tissue outside of the kidney, especially on the anterior surface; the perinephritic abscess was surrounded with indurated tissue, and as, above described, communicated both with the external incision and with the peritoneal cavity. The kidney measured twelve centimetres in length. The left ureter was somewhat dilated, but more noticeably its walls were greatly thickened and very dense. The *right* kidney was enlarged, and presented on its surface and cut section numerous small, yellowish-white streaks and nodules (beginning of acute interstitial nephritis). The pelvis was injected. The ureter was normal.

Bladder: It was contracted, and contained about a teaspoonful of muco-pus; the mucous membrane was of a dark slate-color, with a patch of livid injection on the posterior surface, corresponding to where the cervix of the retroverted uterus had impinged against it. The walls of the bladder were of normal thickness. The openings of the ureters were free and normal. There was a fistulous communication between the posterior surface of the bladder and the vagina on its anterior wall.

Uterus: The uterus was retroverted, and bound down by adhesions of moderate firmness. The posterior lip of the os externum was split, and one portion projected, as a polypoid excrescence, into the vagina. The mucous membrane was smooth and normal. The uterus and vagina were in other respects normal.

The *ovaries rectum* and *liver* were normal.

Stomach: It presented a stellate fibrous cicatrix on its anterior surface.

Intestines normal.

Dr. Lee remarked that he presented this specimen simply because of its surgical interest. It was the only case of cystitis on which he had operated, knowing the patient to be the subject of Bright's disease. He believed the obstinate cystitis justified the operation under the circumstances.

